# Tetrathienothiophene Porphyrin as a Metal-Free Sensitizer for Room-Temperature Triplet–Triplet Annihilation Upconversion

**DOI:** 10.3389/fchem.2022.809863

**Published:** 2022-04-26

**Authors:** Aleksey Vasilev, Anton Kostadinov, Meglena Kandinska, Katharina Landfester, Stanislav Baluschev

**Affiliations:** ^1^ University of Sofia “Saint Kliment Ohridski”, Faculty of Chemistry and Pharmacy, Sofia, Bulgaria; ^2^ Max Planck Institute for Polymer Research, Mainz, Germany; ^3^ University of Sofia “Saint Kliment Ohridski”, Faculty of Physics, Sofia, Bulgaria

**Keywords:** triplet state formation, room temperature, metal-free triplet–triplet annihilation, acidification, inter system crossing

## Abstract

Optically excited triplet states of organic molecules serve as an energy pool for the subsequent processes, either photon energy downhill, such as room temperature phosphorescence, or photon energy uphill process—the triplet–triplet annihilation upconversion (TTA-UC). Manifestation of a high intersystem crossing coefficient is an unavoidable requirement for triplet state formation, following the absorption of a single photon. This requirement is even more inevitable if the excitation light is non-coherent, with moderate intensity and extremely low spectral power density, when compared with the light parameters of 1 Sun (1.5 AM). Coordination of a heavy atom increases substantially the probability of intersystem crossing. Nevertheless, having in mind the global shortage in precious and rare-earth metals, identification of metal-free organic moieties able to form triplet states becomes a prerequisite for environmental friendly optoelectronic technologies. This motivates us to synthesize a metal-free thienothiophene containing porphyrin, based on a condensation reaction between thienothiophene-2-carbaldehyde and pyrrole in an acidic medium by modified synthetic protocol. The upconversion couple tetrathienothiophene porphyrin/rubrene when excited at *λ* = 658 nm demonstrates bright, delayed fluorescence with a maximum emission at *λ* = 555 nm. This verifies our hypothesis that the ISC coefficient in thienothiophene porphyrin is efficient in order to create even at room temperature and low-intensity optical excitation densely populated organic triplet ensemble and is suitable for photon energy uphill processes, which makes this type of metal-free sensitizers even more important for optoelectronic applications.

## Introduction

Optically created molecular triplet organic ensembles serve as an energy pool for numerous succeeding processes and technological applications, such as photocatalytic organic reactions, all-optical sensing of metabolic activity of cell cultures, bio-imaging, and sunlight concentrators for organic solar cells. As an unavoidable prerequisite for all these applications, the requirements to create the organic triplet states at low excitation intensity and at room temperature are stated. The creation of excited triplet states depends drastically on the ISC-parameter of the sensitizer molecule. Furthermore, the ability to relax the accumulated triplet energy *via* an emissive process, such as the phosphorescence, depends also on the ISC parameter: if it is low, no phosphorescence at room temperature is observable. Nevertheless, the process of TTA-UC allows not only to use partially the sensitizer triplet states for an emissive process, such as the delayed fluorescence of the emitter molecules, but also to overcome the destructive influence of the rising sample temperature on the intensity of the emitted light. If the rising sample temperature leads to a significant decrease of the sensitizer phosphorescence, in contrary, the TTA-UC ensures a remarkable increase in the intensity of the delayed fluorescence.

Originally synthesized BODIPY triads (Guo et al., 2014), excited at room temperature by broadband visible light, function efficiently as triplet photosensitizers with enhanced intramolecular resonance energy transfer. Recently (Pristash et al., 2020), heavy-atom–free photon upconversion in a thiosquaraine composite was demonstrated. Hybrid photocatalysts based on C60 molecules, deposited on mesoporous silica, operating at room temperature were demonstrated (Kyriakopoulos et al., 2014). Stable functioning of a heterogeneous photocatalyst (Mori et al., 2013), consisting of iridium and rhodium complexes embedded in a macroreticular acidic resin, was evidenced for visible light–driven H_2_ production. A systematic summary of the advances (Jin et al., 2021) in phosphorescence-based ratiometric sensing of physical parameters and chemical characteristics of living systems, including bioimaging and medical therapy, was recently presented. Iridium(III), ruthenium(II), and rhenium(I) rare-earth metal complexes, phosphorescent at room temperature (Tan et al., 2021) able to monitor the dynamic behavior of subcellular organelles and serving additionally as multifunctional antitumor compounds and integrating imaging and antitumor functions in a single molecule, were reported. Combination of Ir (III)–porphyrins with cell-penetrating and tumor-targeting peptides (Koren et al., 2012) allows sensing the oxygen saturation in various mammalian cell types. Modified phosphorescent materials, combined with amphiphilic or hydrophilic polymers in order to increase their biocompatibility and to allow *in vitro* and *in vivo* bioimaging (Zhen et al., 2021), were recently reviewed. Molecular systems, demonstrating even at highly efficient room temperature thermally activated delayed fluorescence (TADF), were recently (Yang et al., 2017) reviewed. Banu Iyisan (Iyisan et al., 2020) demonstrated minimally invasive and real-time temperature sensing in HeLa cell cultures, based on the process of triplet–triplet annihilation photon energy upconversion, performed in the ambient environment without loss of sensitivity: the sensing organic ensemble was embedded in core/shell nanoparticles, hosting also sacrificial singlet oxygen scavenging oils (FDA-approved as food additives).

Excited organic triplet ensembles interact with the environment much more intensively than the organic singlet states. Therefore, in order to observe bright phosphorescence at room temperature or efficient triplet–triplet annihilation photon energy upconversion (TTA-UC), it is essential to build up a densely populated organic triplet ensemble. In other words, the number of molecules in the excited triplet state should be comparable with the total number of molecules (belonging to the studied species). Importantly, all molecular energy levels, involved in the processes of phosphorescence or TTA-UC should be real molecular levels, i.e., no virtual energetic levels are involved. This structural feature, combined with the relatively long excited state lifetime, allows accumulation of optically excited triplet states as a result of linear absorption of light with very low intensity. Therefore only organic molecules possessing enough high intersystem crossing coefficient (ISC) are able to form an excited triplet state, following absorption of a single photon. Coordination of a heavy atom increases substantially the probability of intersystem crossing. The major experimental demonstrations of densely populated excited triplet ensembles are related to application of precious or rare-earth metals. There are few examples of metal-free TTA-UC (Zhou et al., 2017; Pristash et al., 2020). Nevertheless, having in mind the global shortage in precious and rare-earth metals, identification of metal-free organic moieties able to form triplet states becomes a prerequisite for environmental friendly optoelectronic technologies. This motivates us to synthesize a metal-free thienothiophene containing porphyrin, serving as a sensitizer for the process of TTA-UC.

The process of TTA–UC performed in fluidic organic systems (volatile or nonvolatile organic solvents) or in a hydrophobic soft matter environment relays on optically created densely populated organic triplet ensembles. The driving force for TTA-UC is the intermolecular triplet energy transfer, including the subsequent processes of triplet–triplet energy transfer (TTT) and triplet–triplet annihilation (TTA). All existing TTA-UC models Zhou J et al. (2015); Askes et al. (2017); Gray et al. (2018), state that the annihilation process is diffusion-limited, indirectly assuming the molecular mass transport of excited molecular species as a main physical process behind it, i.e., in order to observe the TTA-UC process, the sensitizer and emitter molecules must be closely arranged; thus, Dexter energy transfer occurs. Therefore in a soft matter matrix, the TTA-UC process demonstrates essential dependences on the material and environmental parameters, such as the degree of overlapping of the interacting energy states, matrix temperature, matrix viscosity, and presence of molecular oxygen, dissolved into the solvent or adsorbed on the soft matter film. It is important to notice that all these materials and environmental parameters are strongly inter-related, and their impact on the densely populated triplet ensembles is not a linear combination of its partial impacts.

Our aim in this regard is to explore the effect of second thienyl core attached to the tetra meso-thienyl function of the porphyrin and to investigate its application as a sensitizer molecule in metal-free TTA-UC systems. To the best of our knowledge, the synthesis of meso-5,10,15,20-tetrathieno[3,2-b]thienyl porphyrin (TTP, Scheme 2) is not reported. Therefore, it was necessary to find the most easy, reliable, and accessible reaction procedures and to adjust the reaction conditions to the needs of the synthetic method of choice.

## Experimental

### Materials and Methods

#### General

Dimethylformamide (DMF), dichloromethane (DCM), acetic acid, propionic acid, triethylamine (TEA), thieno[*3,2-b*]thiophene (**1**), phosphorus oxychloride (POCl_3_), trifluoroacetic acid (TFA), 2,3-dichloro-5,6-dicyano-1,4-benzoquinone (DDQ), *1H*-pyrrole, and glacial acetic acid are purchased from Sigma-Aldrich, TCI Europe or ABCR and are used as supplied. Melting points were determined on a Büchi MP B-545 apparatus and are uncorrected. NMR spectra (^1^H-, ^13^C-NMR) were obtained on a Bruker Avance II + NMR spectrometer operating at 500 MHz for ^1^H- and 125 MHz for ^13^C-NMR using DMSO-d_6_ as a solvent. The chemical shifts are given in ppm (δ) using tetramethylsilane (TMS) as an internal standard. MALDI-TOF mass spectra were recorded on a Bruker iontof TOF.SIMS NCS Mass Spectrometer, at the Max Planck Institute for Polymer Research, Mainz, Germany. The UV-VIS spectra were measured using a Unicam 530 UV-Vis spectrophotometer.

#### Synthesis of thieno[3,2-b]thiophene-2-Carbaldehyde (**2**)

A solution of 14 g (0.1 mol) of thieno[*3,2-b*]thiophene in 22 ml DMF was cooled in ice water. To the solution, 15.3 g (0.1 mol) of phosphorus oxychloride was added during 15 min, followed by vigorous stirring. During the addition of phosphorous oxychloride, the temperature in the reaction vessel was not allowed to exceed 20°C. The reaction mixture was stirred for 15 min at 0°C, 30 min at room temperature, and then heated to 60–65°C, at which temperature an exothermic reaction started. When the exothermic reaction had ceased, the reaction mixture was heated for 45 min on a steam bath, cooled, and poured on ice. The pH was adjusted to 5–6 with solid sodium acetate, and the reaction mixture was left overnight. The reaction product was extracted with ether, and the ether was washed with water, then with saturated sodium bicarbonate solution, and dried with anhydrous magnesium sulfate. Yield 81%; ^1^H-NMR (500 MHz, CDCl_3_; δ (ppm): 7.36 d (1H, CH, ^3^J_HH_ = 5.0 Hz); 7.73 d (1H, CH, ^3^J_HH_ = 5.3 Hz); and 7.97 s (1H, CH), 10.00 s (1H, CHO). ^13^C NMR (135 MHz, CDCl_3_ δ(ppm)): 120.19, 129.09, 133.90, 139.22, 145.49, 145.76, and 183.59. Mp = 54–56°C (lit. 52–55°C).

### Synthesis of 5,10,15,20-tetrakis(thieno[3,2-b]thiophen-2-yl)porphyrin (**TTP**)

#### Procedure A

In a 50-ml round bottom flask, equipped with an electromagnetic stirrer and a reflux condenser, 0.5 g (0.0297 mol) thieno[*3,2-b*]thiophene-2-carbaldehyde (**2**), 0.21 g (0.00312 mol, 0.22 ml) *1H*-pyrrole (**3**) and 10 ml glacial acetic acid were added, and the mixture was heated at 60°C for 3 h. The reaction mixture was cooled to room temperature, and 20 ml methanol was added. The formed precipitate was suction filtered and washed with methanol three times. The residue was purified on a short path silica column with eluent TEA: n-heptane: DCM = 0.2: 2: 3. The product was isolated as a first position on the column. The analytical sample was obtained after subsequent recrystallization from DCM: MeOH. Yield: 0.051 g (2%). ^1^H-NMR (500 MHz, CD_2_ClCD_2_Cl; d, ppm): 2.64 s (2H, NH); 7.54 d (4H, CH, ^3^J_HH_ = 5.2 Hz); 7.66 d (4H, CH, ^3^J_HH_ = 5.0 Hz); 8.11 s (4H, CH); and 9.15 s (8H, CH). ^13^C NMR DEPT (135 MHz, CD_2_ClCD_2_Cl δ(ppm)): 112.8 CH, 119.3 CH, 126.2 CH, 127.4 CH, 138.7 CH, 141.6 CH, and 144.3 CH (MALDI-TOF (*m/z*): found 861.9509, calcd 861.96 for C_44_H_22_N_4_S_8_. IR (nujol) ν_max_: 2395, 2305, 1190, 1110, 1495, 1340, 1190, and 580 cm^-1^; and UV–vis [toluene, *λ*_max_ (nm), *ε* (l.mol^-1^. cm^-1^]: 437 (152000), 526 (8990), 567 (5390), 597 (3880), and 665 (2510)].

#### Procedure B

In a 50-ml round bottom flask, equipped with an electromagnetic stirrer and a reflux condenser, 0.5 g (0.0297 mol) thieno[*3,2-b*]thiophene-2-carbaldehyde (**2**), 0.21 g (0.00312 mol, 0.22 ml) *1H*-pyrrole (**3**), and 10 ml glacial acetic acid were added, and the mixture was heated at reflux for 30 min. The reaction mixture was cooled to room temperature, and 20 ml methanol was added. The formed precipitate was suction filtered and washed with methanol three times. The residue was purified on a short path silica column with eluent TEA: n-heptane: DCM = 0.2: 2: 3. The product was isolated as a first spot. The analytical sample was obtained after subsequent recrystallization from DCM: MeOH. Yield: 0.063 g (2.4%).

#### Procedure C

In a 50-ml round bottom flask, equipped with an electromagnetic stirrer and a reflux condenser, 0.5 g (0.00297 mol) thieno[*3,2-b*]thiophene-2-carbaldehyde (**2**), 0.21 g (0.00312 mol, 0.22 ml) *1H*-pyrrole (**3**), and 10 ml propionic acid were added, and the mixture was heated at reflux for 1 h. The reaction mixture was cooled to room temperature, and 20 ml methanol was added. The formed precipitate was suction filtered and washed with methanol three times. The residue was purified on a short path silica column with eluent TEA: n-heptane: DCM = 0.2: 4: 1. The product was isolated as a first spot by TLC. The analytical sample was obtained after subsequent recrystallization from DCM: MeOH. Yield: 0.085 g (3.3%).

#### Procedure D

In a 1000-ml, round bottom flask, equipped with an electromagnetic stirrer, 1 g (0.00684 mol) thieno[*3,2-b*]thiophene-2-carbaldehyde (**2**), 0.47 ml (0.46 g, 0.00684 mol), and *1H*-pyrrole (**3**) were dissolved in 500 ml dry dichloromethane. Then, 0.35 ml (0.52 g, 0.0046 mol) trifluoroacetic acid (TFA) was added dropwise by syringe for about 15 min, and the reaction solution was vigorously stirred at room temperature for 4 h in the dark. Furthermore, 2.64 g (0.01163 mol) of DDQ was added, and the mixture was stirred for 30 min at the same conditions. In addition, 4 ml of triethylamine was added, and the solvent was removed using a rotary evaporator to volume of 15 ml. To the dark-colored residue, 30 ml methanol was added. The precipitate was suction filtered and washed with methanol. The analytical sample was obtained after silica flash column chromatography with eluent ethyl acetate: petrol ether = 3:2. Yield: 0.03 g (5.1%).

### TTA-UC

**FIGURE 1:**
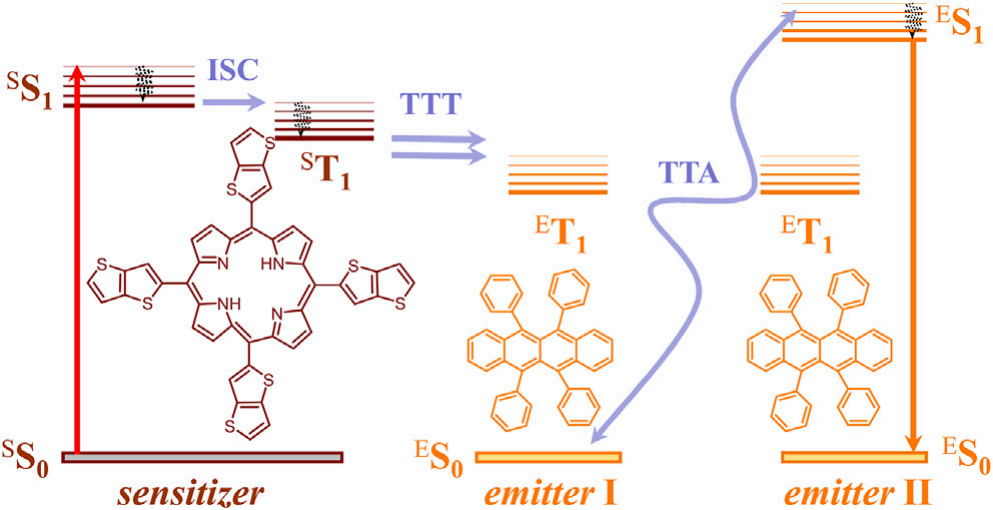
Simplified energetic scheme of the TTA−UC process in the multicomponent organic system in a molecular oxygen-free environment. Left—structures of the sensitizer (TTP, *meso*-5,10,15,20-tetrathieno[*3,2-b*]thienyl porphyrin) and right—structure of the emitter (rubrene).

The process of TTA-UC is the only upconversion process realized up to now with non-coherent, ultra-low intensity sunlight (Baluschev et al., 2006). A key advantage of the TTA-UC using techniques is that the conversion processes and devices can be considered and optimized independently, without affecting the particular properties of the operating photochemical material or device architecture. The TTA-UC takes place in multi-chromophore systems consisting of energetically optimized pairs of emitter molecules (typically aromatic hydrocarbons) and sensitizer molecules (usually, metalled macrocycles, such as porphyrins and phthalocyanines), as shown in Figure 1. Shortly, the photon energy absorbed by a sensitizer is stored in its metastable triplet state, formed in the process of intersystem crossing (ISC). Furthermore, this energy is transferred to an emitter triplet state *via* the process of TTT. Next, the excited triplet states of two emitter molecules undergo TTA, in which one emitter molecule returns back to its singlet ground state and the other molecule gains the energy of both triplet states and is excited to the higher singlet state. As the singlet state emitter decays radiation back to the ground state, a delayed fluorescence photon (the blue arrow, Figure 1) bearing a higher energy than that of the excitation photons is emitted.

## Results and Discussion

### Synthesis

Functionalized thienyl-appended porphyrins are significant compounds in many high technology applications, owing to their important physicohemical properties: in a recent comprehensive review, Boyle et al., (2010) demonstrated applications of meso-tetrathienylporphyrins, based on its convenient preparation and ease of functionalization, allowing a large variety of energy transfer reactions, excellent film-forming and pronounced electro-conductivity behavior.

**SCHEME 1:**
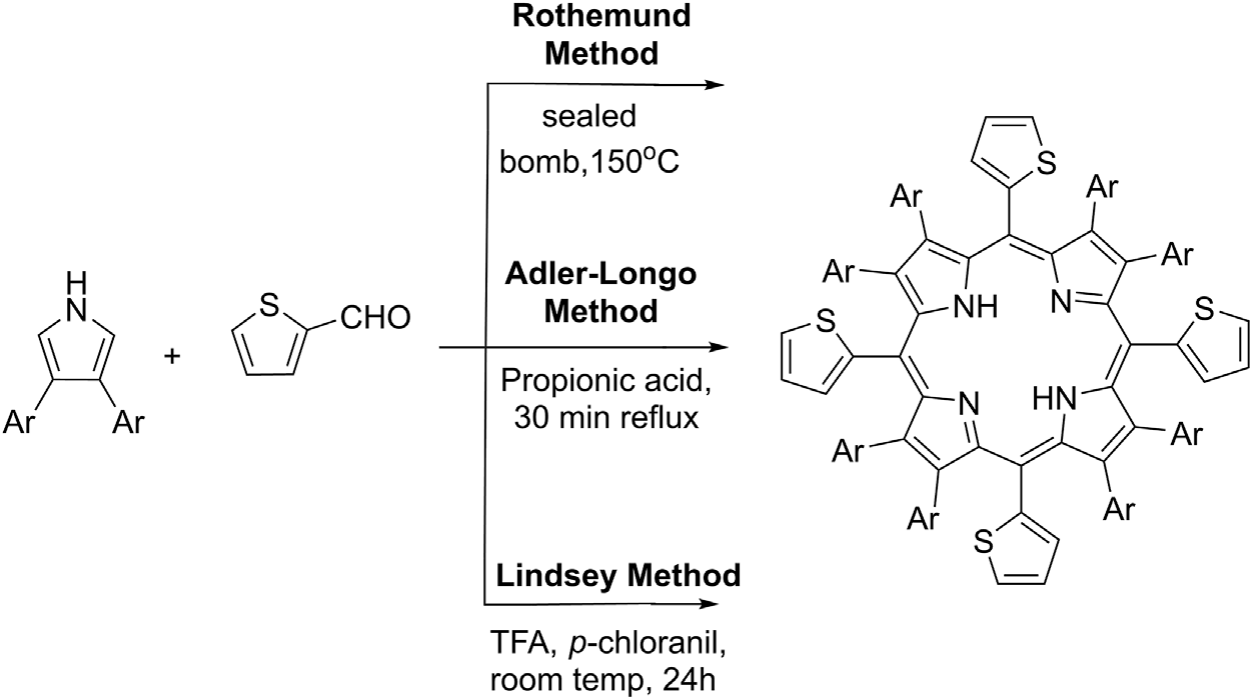
Synthetic methods and the general structure of substituted thienyl-porphyrins.

Different modifications of the main synthetic methods demonstrated on Scheme 1 for the preparation of meso-5,10,15,20-thienyl porphyrins and their derivatives were reported in the literature. The first reported synthesis of meso-tetra (thien-2-yl)porphyrin (Scheme 1) was presented by Triebs and Haeberie et al. (1968) by the condensation of pyrrole and 2-thiophenecarboxaldehyde by a modified Rothemund method giving 9% yield. The Adler-Longo’s method was later applied (Torrens et al., 1972) by allowing the reaction of equimolar amounts of 2-thiophenecarboxaldehyde and pyrrole in refluxing propionic acid, but the reaction yields were not reported (Scheme 1). The main efforts to date were focused to adapt the so-called Lindsey’s method for the synthesis of the commented here type of porphyrins (Scheme 1). Lindsey’s conditions are much milder in comparison to previous methods. Normally, such Lindsey-type synthesis requires room temperature, dehydrogenation reagent (such as p-chloranil or DDQ), and acid. The mixture is subsequently treated with a basic reagent. Thus, Lindsey’s method for the preparation of porphyrins provides higher reaction yields and higher purity of the crude reaction products (Lindsey et al., 1987; Ono et al., 1998). Using the commented synthetic strategies, a large variety of polythiophenyl-substituted porphyrins were synthesized and their photophysical and electrochemical properties have been explored (Boyle et al., 2010).

**SCHEME 2:**
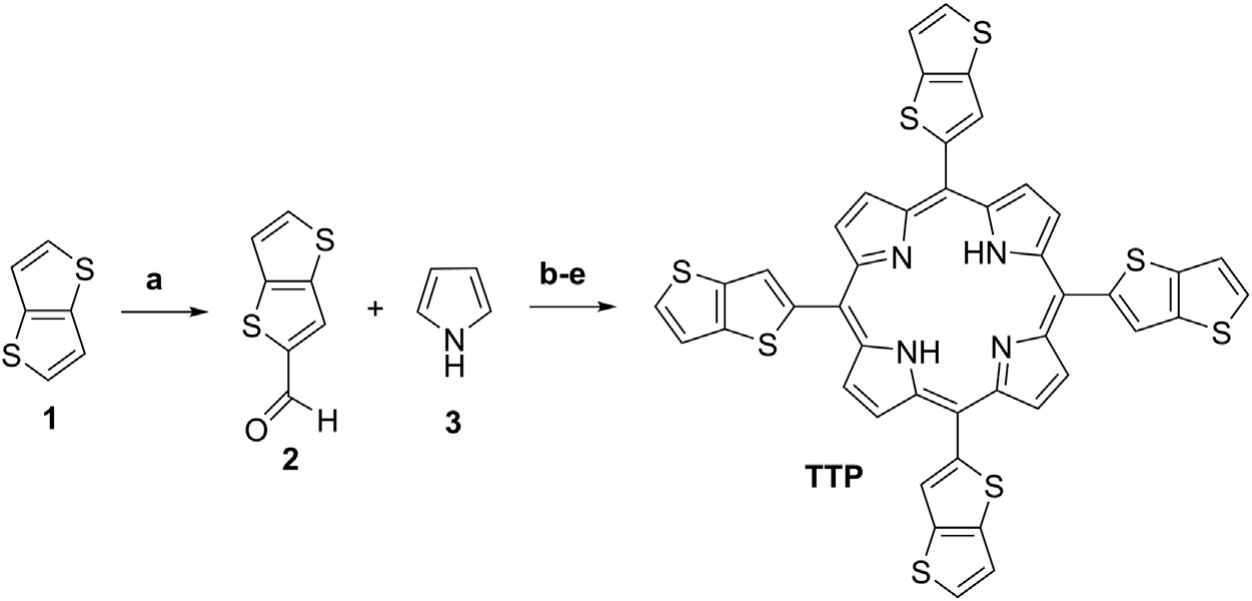
Reaction conditions and yields: **(A)** Preparation of intermediate 2: 1, DMF, POCl3, 80°C, 2 h, and Yield: 81%; **(B)** Adler’s method procedure A: 2, 3, acetic acid, 60°C, 2 h, and Yield: 2%; **(C)** Adler’s method procedure B: 2, 3, acetic acid, reflux 30 min, and Yield: 2.4%; **(D)** Adler’s method procedure C: 2, 3, Propionic acid, reflux 1 h, 3.3%; and **(E)** Lindsey’s method procedure D: 2, 3, r. t., dry DCM, TFA, DDQ, TEA, and Yield: 5.1%.

First, the starting thieno[3,2-b]thiophene-2-carbaldehyde (2) (Scheme 2) was prepared by the Vilsmeier–Haack-Arnold formylation reaction of thieno[3,2-b]thiophene in DMF as a reagent and as a reaction media in the presence of a slight molar excess of phosphorus oxychloride (Bugge, 1971). The structure of the compound was proved by thin-layer chromatography (TLC), ^1^H-NMR, 13C-NMR spectroscopy, and observing the melting point. For the synthesis of the target meso-5,10,15,20-tetrathieno[3,2-b]thienyl porphyrin (Scheme 2), a large number of reaction conditions were examined by varying the reaction temperature, changing the solvents, and the molar quantities of the reagents and the reaction time as well.

Herein, we described the reaction conditions ensuring the best reaction yields in our hands. In a series of experiments, the best molar quantities for the reactants ensuring optimal reaction yields were observed. The most promising procedures with the optimal molar quantities of the starting materials are based on Adler-Longo’s method and Lindsey’s method (Scheme 2). In procedure A, was used glacial acetic acid for reaction media because of the lowest boiling temperature in comparison with the propionic acid. The mild reaction temperature at 60°C does not lead to higher yields but significantly decreases the amounts of tarry side products formed during the reaction, thus providing facile purification.

Heating the reaction mixture at 120°C for 30 min afforded a slight increase in the reaction yield (Scheme 2C, Procedure B). Changing the solvent to propionic acid and ensuring 1 h reflux (141°C) in an open-air system afforded 3.3% reaction yield (Procedure C, Adler-Longo’s method). Finally, by modifying Lindsey’s procedure by leading the reaction in an optimized volume of dry DCM in the presence of TFA and using the milder dehydrogenating reagent DDQ instead of p-chloranil (classical Lindsey’s conditions), we obtained the higher for the given compound reaction yield (5.1%) with the best purity among the mentioned ones (Scheme 2E, Procedure D). The chemical structure of the target porphyrin TTP was proved by several analytical methods. The proton NMR spectra demonstrated typical for the meso-tetrathienyl porphyrins signals for the aromatic protons (Bhyrappa and Bhavana, 2001). Typically, the imine protons NH from the porphyrin cycle appear as a singlet with integral intensity for two protons at -2.64 ppm (Supplementary Material). Two doublets at 7.53 and 7.65 with an integral intensity for four protons each and with J-constants around 5 Hz can be assigned to the thienyl protons from the theino[3,2-b]thiophene functionality. The singlet at 8.11 ppm with the integral intensity corresponding to four protons is assigned to the CH-proton from the theino[3,2-b]thiophene moiety as well. Typically for the unsubstituted pyrrole part of the meso-substituted porphyrin, the broad singlet appeared at 9.15 ppm with an integral intensity for eight protons. The carbon NMR spectra (SI), the UV-VIS spectra, and the MALDI-TOF spectra unequivocally prove the chemical structure and the purity of the final compound TTP (Supplementary Material). The relation between the chemical structure and the photo-physical properties of the target porphyrin TTP is described in the next section.

### Optical Properties

**FIGURE 2:**
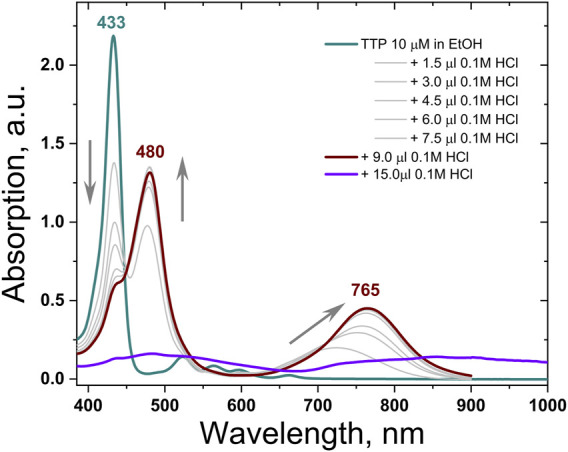
Absorption spectra of TTP in ethanol neat (the dark cyan line) and in the presence of 3 × 10^–4^ M HCl (the wine line) and in the presence of 5 × 10^–3^ M HCl (the violet line). For clarity, all intermediate absorption spectra are in light gray.

The UV-VIS absorption spectra of TTP demonstrate the typical for all *meso-*tetrathienyl porphyrin absorption bands. The highly intensive absorption Soret-band is attributed to the higher energy S_0_→S_2_ electron transitions, and it appears at *λ* = 433 nm followed by four quite lower intensity Q-bands relative to S_0_→S_1_ electron transitions (Figure 2, the dark cyan line). The red shifted band in the absorption spectra of TTP at *λ* = 665 nm is bathochromically shifted in comparison with the same in the regular *meso*-tetrathienyl porphyrins *λ* = 613 nm (Zhou Y et al., 2015) probably due to the enhanced electron-donating properties of the thienothienyl function in comparison with thien-2-yl one. Titration of ethanol solution of TTP with successive steps of 0.1 M HCl demonstrates pronounced halochromic behavior: the Soret-band of the porphyrin at *λ* = 437 nm decreases substantially; simultaneously, a new absorption band grows up with a central wavelength at *λ* = 480 nm. Additionally, stepwise appearance and monotonic increase of the intensity of the absorption Q-band with the central wavelength at *λ* = 760 nm (Figure 2, the wine line) was observed (up to 3 × 10^–4^ M HCl). Further increase of acidity leads to a dramatic decrease of the total absorption (Figure 2, the violet line).

In a strong acid media, the Q-band and S-band of the thienothienyl porphyrins shift bathochromically; additionally, the multiple Q-bands of the free base thienothienyl porphyrin grow together and re-build in an intensive and substantially red shifted Q-band (for more than Δλ = 100 nm). This demonstrates that the absorption spectrum of the studied TTP can be tuned significantly toward the near IR spectral range, without any additional synthetic steps. The direct demonstration of TTA-UC in an acidic medium was not possible since the used emitter rubrene is not directly soluble in ethanol. We intend to decorate emitter molecules in order to increase their solubility in such media and to observe TTA-UC.

Supplementary Figure S6 (SI -part) shows the fluorescence spectrum of the studied TTP-sensitizer in strong acidic media, excited at *λ*_exc_ = 785 nm. This wavelength is completely outside the TTP spectrum in hydrophobic media (Figure 2, the dark cyan line).

The emitter molecule rubrene (Figure 1) possesses a very broad excited triplet state, having energies between 1.04 and 1.29 eV (Liu and Larry, 1977). As mentioned earlier, metal-free TTP does not show phosphorescence at room temperature. We studied the TTA-UC efficiency of the metal-free TTP by testing different perylene and perylene monoimide derivatives, working well with mixed benzo-naphtho and tetranaphtho porphyrins (Heinrich et al., 2018). However, the studied metal-free TTP works efficiently only with rubrene: therefore, its triplet state can be comparable with those of tetraanthraporphyrins, i.e., 0.95 eV.

The TTA-UC process suffers doubly by the presence of even a small concentration of molecular oxygen; first, corresponding to the oxygen concentration and/or the rate of molecular oxygen diffusion throughout the sealing materials, significant amounts of optically excited sensitizer triplet states are lost, and second (which is an even more destructive consequence), a considerable amount of the photoactive molecules are being damaged, and they fail out from the UC process. Linear polyunsaturated triterpene, such as squalene (Shimizu et al., 2018), is proved to be a very efficient sacrificial singlet oxygen scavenger (SSOS). This SSOS material introduces a huge excess of “sacrificial” double bonds, combined with low viscosity (at room temperature), essential for high molecular mobility of the UC molecules, ensuring relatively high efficiency of the TTA-UC process.

**FIGURE 3:**
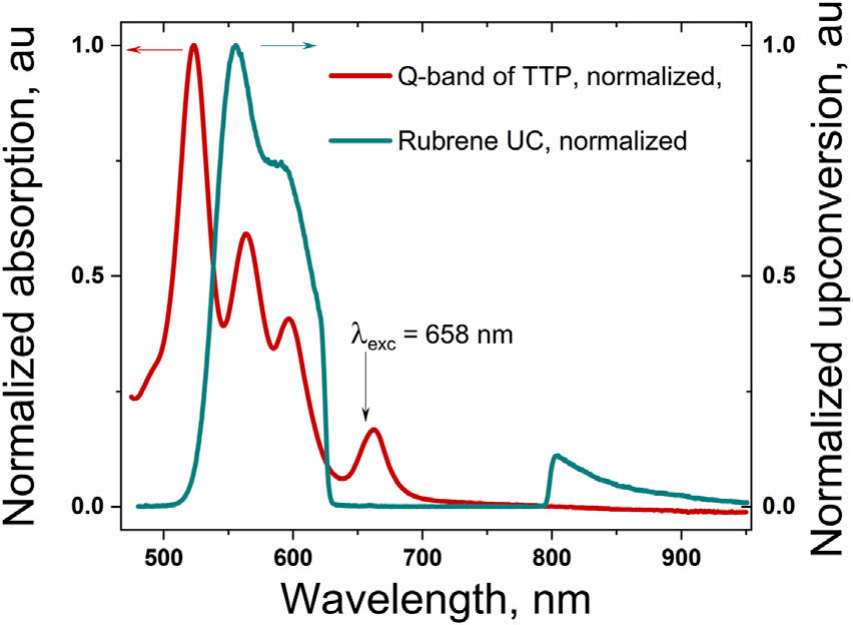
Normalized Q-band absorption spectrum of the meso-5,10,15,20-tetrathieno[3,2-b]thienyl porphyrin, dissolved in 98 toluene/2 vol% squalene (the dark red line); normalized delayed fluorescence of rubrene (the cyan curve) excited in UC regime, whose excitation wavelength *λ*_exc_ = 658 nm. The excitation wavelength is suppressed by using a super broadband notch filter (rejection: more than 10^4^ for the wavelength range centered at Δλ = 705 nm, with FWHM = 175 nm, and transparency, for all other wavelengths, better than 0.99).

Figure 3 shows the normalized Q-band absorption spectrum of the TTP, dissolved in a mixture of toluene and squalene. The sample is prepared and sealed in a nitrogen-filled glove box (residual oxygen concentration, 2ppm). The addition of 2 %vol. of squalene ensures the complete scavenging of the residual molecular oxygen; thus, the UC sample could be defined as oxygen-free.

The quantum yield $\left(Q.Y._{TTA-UC}\right)$ of the TTA-UC process is certainly of decisive importance. The IUPAC definition of quantum yield (Q.Y.) for the arbitrary emissive process defines it as a ratio of the number of emitted photons to the number of absorbed photons. It has to be pointed out explicitly that such a classical term (i.e., $Q.Y._{TTA-UC}=N_{emitted}^{photons}/N_{absorbed}^{photons}$ ) is attributed to a complex process such as the diffusion controlled TTA-UC (consisting of a chain of intramolecular and intermolecular energy transfer processes, namely, ISC, TTT, TTA, and diffusion of excited triplet states). Here, $N_{absorbed}^{photons}$ represents the number of photons absorbed by the UC sensitizer molecules, and $N_{emitted}^{photons}$ represents the number of photons emitted by the UC emitter molecules. The main outcome of such a simplified definition is that clear and noncontradictive knowledge about a real UC photon flux is expected for the given excitation conditions.

**FIGURE 4:**
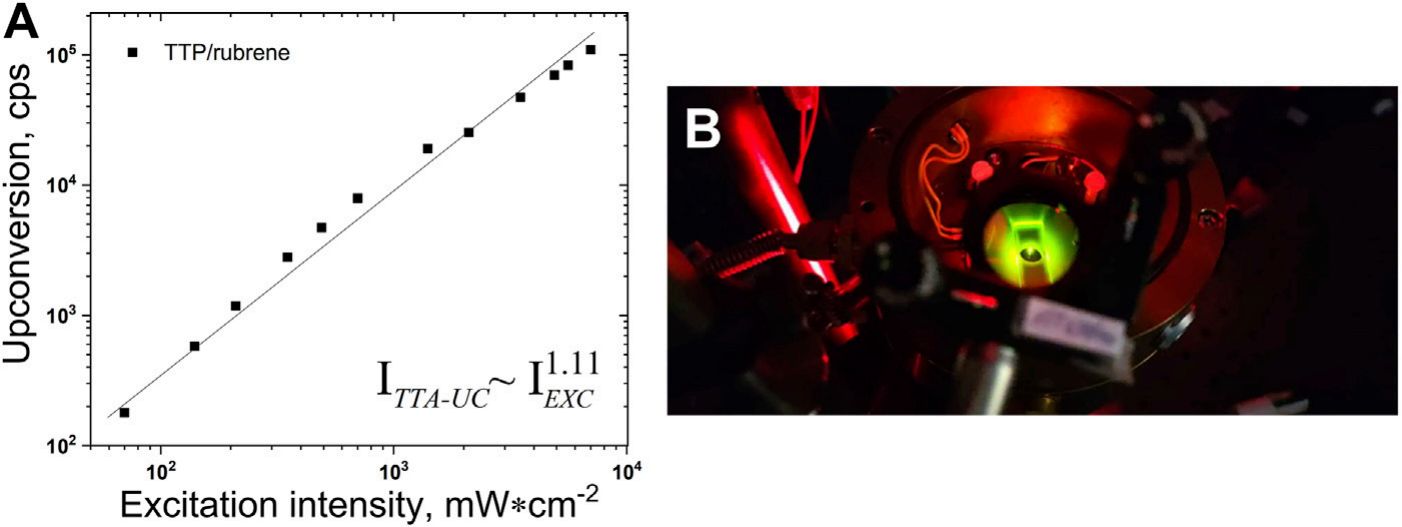
**(A)**–Dependence of the UC intensity on the excitation intensity. *Experimental conditions*: UC couple, TTP (2 × 10^-5^ M)/rubrene (4 × 10^-4^ M); ratio sensitizer/emitter - C_S_/C_E_ = 1/20; room temperature; sample thickness – 1000 μm; single-mode laser diode, *λ*_exc_ = 658 nm; optical registration–*via* fiber–spectrometer (C10083CA, Hamamatsu Inc.) with absolute wavelength calibration and corrected spectral response; integration time, 100 ms; excitation beam diameter, d_EXC_ = 1600 µm; Vitrotube^®^ glass sample, sealed in a nitrogen-filled glove-box, residual oxygen < 2 ppm; each measurement is performed on a neat sample spot; solvent, 98 vol% toluene/2 vol% squalene; Q.Y._TTA-UC_ = 0.018 (following the IUPAC definition). **(B)**–Photograph of a working metal-free TTA-UC sample. In order to suppress the diffuse scattered excitation light, a super broadband notch filter was used.

The sub-quadratic dependence of the UC fluorescence on the excitation intensity, demonstrated for many UC systems (Baluschev et al., 2016; Askes and Bonnet, 2018) dissolved in organic solvents in oxygen-free conditions is reproduced here for the UC systems dissolved in 98 vol% toluene/2 vol% squalene (Figure 4). The experimental data are well approximated with a power law $I_{TTA-UC}=a\times I_{exc}^{b}$ , where the parameter **b** = 1.11.

## Conclusion

We demonstrated the effective formation of densely populated optically excited triplet ensembles, by means of metal-free thienothiophene-containing porphyrins operating at room temperature and low excitation intensity (as low as 100 mW×cm^-2^). Furthermore, we observed efficient triplet–triplet annihilation upconversion with Q.Y. as high as 0.018, following the IUPAC definition for quantum yield. Manifestation of a high intersystem crossing coefficient is an unavoidable requirement for triplet state formation, following absorption of a single photon. We synthesized a metal-free thienothiophene-containing porphyrin based on a condensation reaction between thienothiophene-2-carbaldehyde and pyrrole in an acidic medium. This proves that thienothiophene porphyrins exhibit ISC coefficients high enough to perform photon energy uphill processes, which makes this type of metal-free sensitizers even more important for optoelectronic applications.

## Data Availability

The original contributions presented in the study are included in the article/Supplementary Material, further inquiries can be directed to the corresponding authors.
