# Supplementary material for: Tetrathienothiophene Porphyrin as a Metal-Free Sensitizer for Room-Temperature Triplet–Triplet Annihilation Upconversion
Source: Front Chem. 2022 Apr 26;10:809863. doi: 10.3389/fchem.2022.809863 (PMC9086237; doi:10.3389/fchem.2022.809863)
Supplement: Supplementary file 1 [file DataSheet1.docx]

**Supplementary Materials**

Tetrathienothiophene porphyrin as metal-free sensitizer for room-temperature triplet-triplet annihilation upconversion

Aleksey Vasilev^1, 2^, Anton Kostadinov^2^, Meglena Kandinska^1^, Katharina Landfester^2^*, Stanislav Baluschev^2, 3^*

^1^ University of Sofia “Saint Kliment Ohridski”, Faculty of Chemistry and Pharmacy, 1 James Bourchier blvd., Sofia 1164, Bulgaria;

^2^ Max Planck Institute for Polymer Research, Ackermannweg 10, Mainz 55128, Germany

^3^University of Sofia “Saint Kliment Ohridski”, Faculty of Physics, 5 James Bourchier blvd*.,* Sofia 1164, Bulgaria.

*** Correspondence:**[landfester@mpip-mainz.mpg.de](mailto:landfester@mpip-mainz.mpg.de); [balouche@phys.uni-sofia.bg](mailto:balouche@phys.uni-sofia.bg)

**Figure S1a**. Full range (0-10 ppm) ^1^H-NMR spectra of thieno[*3,2-b*]thiophene-2-carbaldehyde **2**

**Figure S1b**. Aromatic area (7-10 ppm) of ^1^H-NMR spectra of thieno[*3,2-b*]thiophene-2-carbaldehyde **2**.

**Figure S2**. ^13^C-DEPT-NMR spectra of thieno[*3,2-b*]thiophene-2-carbaldehyde **2**.

**a**

**b**

**Figure S3:** ^1^H-NMR spectra of *Meso*-5,10,15,20-tetrathieno[*3,2-b*]thien-2-yl-porphyrin (**TTP**). (**a**) Complete ^1^H-NMR spectra of **TTP**; (**b**) Aromatic part from ^1^H-NMR spectra of dye **TTP**.

**Figure S4:**  ^13^C-DEPT-NMR spectra of *Meso*-5,10,15,20-tetrathieno[*3,2-b*]thien-2-yl-porphyrin (**TTP**).

**a**


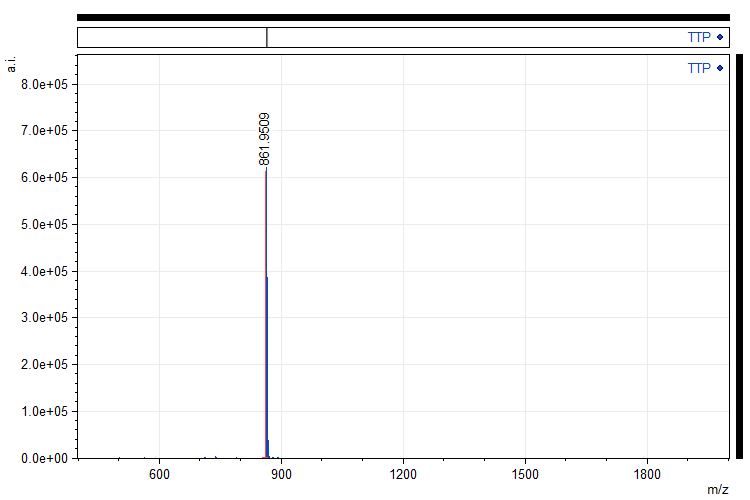


**b**

**Figure S5:** (**a**) MALDI-TOF spectra of *Meso*-5,10,15,20-tetrathieno[*3,2-b*]thien-2-yl-porphyrin (**TTP**); (**b**) Calculated by ChemDraw Exact Mass of **TTP**.


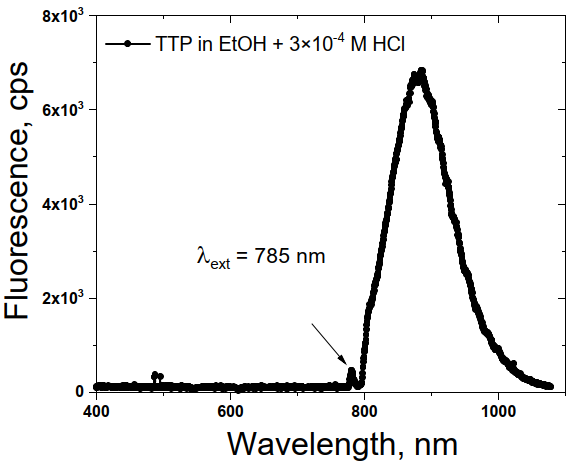


**Figure S6:** Fluorescence spectrum of metal-free TTP in the presence of a strong mineral acid. **Experimental conditions**: TTP - 1×10^-6^M; room temperature; sample thickness – 1000 µm; single mode laser diode, λ_exc_ = 785 nm; excitation beam diameter - d_EXC_ = 1600µm; Vitrotube® glass sample, sealed in nitrogen filled glove-box, residual oxygen < 2ppm; solvent – ethyl alcohol + 3×10^-4^M HCL (hydrochloric acid); Q.Y._F_ = 0.11 (following the IUPAC definition).


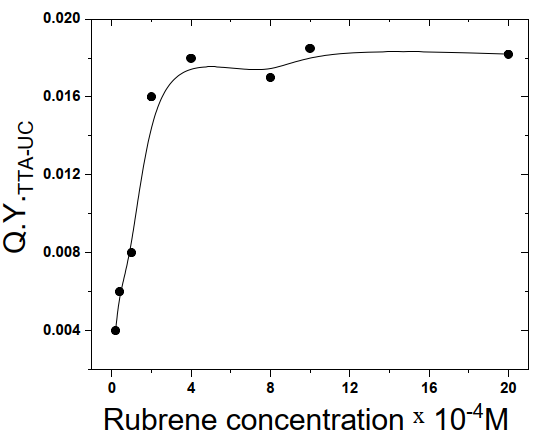


**Figure S7**: Dependence of the quantum yield of the TTA-UC couple TTP/rubrene on the emitter molar concentration. ***Experimental conditions***: sensitizer concentration TTP - 2×10^-5^M; emitter concentration – shown in the graph; the ratio sensitizer/emitter - C_S_ / C_E_ = is changed gradually, starting with 1/1 up to 1/100; room temperature; sample thickness – 1000 µm; single mode laser diode; λ_exc_ = 658 nm; optical registration – *via* fiber spectrometer; excitation beam diameter – d_exc_ = 1600µm; excitation intensity – 200mW×cm^-2^; Vitrotube® glass sample, sealed in nitrogen filled glove-box, residual oxygen < 2ppm; solvent – 98 vol% toluene / 2 vol% squalene.

**Table TS1**.

| **Excitation intensity,** mW×cm^-2^ | **UC delayed fluorescence**, cps |
| --- | --- |
| 70 | 179 |
| 140 | 579 |
| 210 | 1186 |
| 350 | 2777 |
| 490 | 4696 |
| 700 | 7931 |
| 1400 | 18998 |
| 2100 | 25415 |
| 3500 | 47204 |
| 4900 | 69949 |
| 5600 | 82908 |
| 7000 | 108896 |


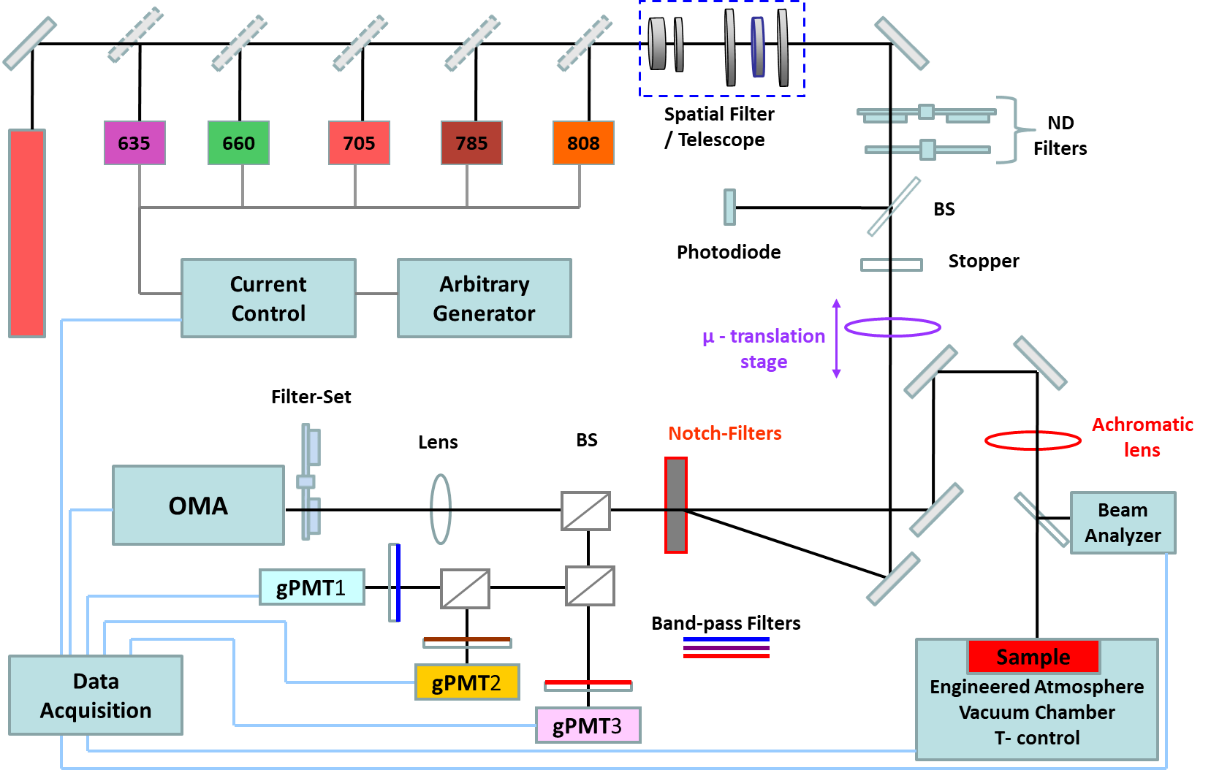


**Figure S8**: Set-up for registration of the dynamical parameters of the TTA-UC in engineered atmosphere with actively controlled oxygen content. Depending on the excitation laser wavelength the central wavelength of the notch filter is changed accordingly. There is possibility to measure – (***i***) sealed liquid samples – at atmospheric pressure of 1 bar; – (***ii***) not sealed samples – films, in vacuum chamber with 1×10^-5^ torr dynamical pressure or engineered atmosphere. The samples are measured in environment with active temperature control and temperature stabilization. In order to minimize the effect of solvent convection, the liquid samples are measured in horizontal position.

The set-up for registration of the dynamical parameters of the TTA-UC in engineered atmosphere with actively controlled oxygen content is shown in Figure S8. As excitation sources temperature & current stabilized, single mode continuous-wave (*cw)*-diode lasers, mounted on laser heads (TCLDM9, *Thorlabs Inc*.) and operating at different wavelengths were used (Figure 8.4). All lasers are collimated to a mutually uniform spot with aspect ratio of ~ 1.1 by applying cylindrical telescopes. The temperature & current control of all diode lasers is performed by PRO8000 / 8-Slot Modular Rack Chassis (*Thorlabs Inc*.), supporting laser current control units LDC8002 / LDC8010 and laser diode temperature controller modules TED8040 (*Thorlabs Inc*.).

Using single-edge short-pass dichroic beam-splitters with proper spectrum is possible to combine any two laser wavelengths, demonstrated at Figure 8.4. For instance, applying the short-pass dichroic beam-splitter FF697-SDi01-25x36 (*Semrock Inc*.) it is possible to combine at the same propagation axes the lasers with (**i**) λ=635 nm / λ=705 nm; or (**ii**) λ=635 nm / λ=785 nm; as well as (**iii**) λ=660 nm / λ=705 nm.

**Each laser beam** pass through the spatial filter in order to reach nearly TEM_00_ transversal intensity distribution. A series of reflective neutral density (ND) filter (*Thorlabs Inc.*) placed on revolving optical holder (in order to keep the optical axes of the experiment unchanged) were used to attenuate smoothly the beam power. Afterward, the beam passed through the system of ultra-broadband mirrors (MaxMirror*, Semrock Inc.*) and finally focused by achromatic lens (NA=0.24) onto the sample. A defocusing achromatic lens (the violet lens, Figure S8) placed on an electronically controlled µ-positioning stage (DL125 Delay Line Stage, *Newport Corp.*) controls the excitation spot diameter. The resulting excitation spot diameter is permanently controlled by a beam profiler (BP104-VIS*, Thorlabs Inc*.). The optical density of the all samples at the excitation wavelength is nearly 0.1; therefore the attenuation of the pump light can be neglected for the given thickness of the optical samples (1000 µm or 400 µm). The luminescence emission generated by the sample was collected with the same apochromatic lens, thus the excitation- and observation- spots are completely spatially overlapped. The emission of the excitation lasers was rejected by the appropriate notch filters: for instance by application of a notch filter designed for λ=633 nm (FWHM ~ 29nm, NF 03-633E-25, *Semrock Inc*.) rejection better than 10^6^ times can be achieved. Other notch filters are λ=658 nm (NF03-658E-25, *Semrock Inc*.), λ=785 nm (NF03-785E-25, *Semrock Inc*.) and λ=808 nm (NF03-808E-25, *Semrock Inc*.).

**The *cw* - emission spectra** were registered by Optical Multichannel Analyzer (*Hamamatsu Photonics Inc*.) or fiber – spectrometer (C10083CA, *Hamamatsu Photonics Inc*.) with absolute wavelength calibration and corrected spectral response.

**For the time-resolved measurements** of the sensitizer residual phosphorescence and delayed emitter fluorescence additional removable beam splitters were installed in order to reflect the sample emission toward the time-resolved registration unit. The used laser heads (TCLDM9, *Thorlabs Inc*.) allow current modulation *via* modulation input, depending on the maximal current supplied, the modulation frequency vary from 50 kHz up to 200kHz. Therefore, the laser intensity was modulated by arbitrary pulse generator (TGA1244, *TTi Inc.*) with caring frequency of 40 MHz. The obtained laser diode pulses have almost rectangular time-shape, with rise- and decay- slopes less than 2.5×10^-6^s.

The time-response function of the used set-up is better than 50×10^-9^s which is much lower than the characteristic time scale of the processes of TTA and TTT (usually, in order of 10 ÷ 100×10^-6^ s). The time interval between the excitation pulses can be produced to be large enough, no technical limits apply (arbitrary pulse generator is used). Therefore, the cross-talk between the optically excited molecular ensembles is completely avoided. One edge – beam splitter (FF01-694/SP-25, *Semrock Inc*.) was used to separate spatially the sensitizer residual phosphorescence and delayed emitter UC-fluorescence, registered by gPMT1 and gPMT2, accordingly. Additional edge-filters, namely a short pass (SP01-633RS-25, *Semrock Inc*.) filter with cutting wavelength λ = 650 nm and long pass (HQ700LP, *Chroma GmbH*) filter with cutting wavelength λ = 700 nm, were installed in front of the registration opto-electronic devices at the delayed-fluorescence branch and phosphorescence branch, respectively.

The optical signals were detected by gated photo-multiplier tubes H1156-01-NF (gPMT1 and gPMT2, Figure S8*, Hamamatsu Photonics Inc*.) and gated photo-multiplier tube H1156-20-NF with extended red multialkali photocathode with enhanced sensitivity (gPMT3, Figure 8.4*, Hamamatsu Photonics Inc.*). The gPMT’s are working in analogous mode with a rise- and decay- times less than 30×10^-9^ s. The gated photo-multiplier tubes are set in “Normally OFF” mode. The gating pulses should be with amplitude of 3.5V ÷ 5V applied on 10kΩ load, with maximal repetition rate of 10 kHz up to ∞. The gPMT-operation delay is less than 0.2×10^-6^ s. Each gPMT is equipped with a wide bandwidth amplifier unit C9663 (DC up to 150MHz, *Hamamatsu Photonics Inc.*), where the current input signal from the photomultiplier tube is directly converted into a voltage output signal with 50 Ω load at conversion factor of 4mV/µA. The electrical signals were registered by multi-channel digital storage oscilloscope (54622A, *Agilent Inc*.) with a bandwidth of 100 MHz and 12 bit resolution.


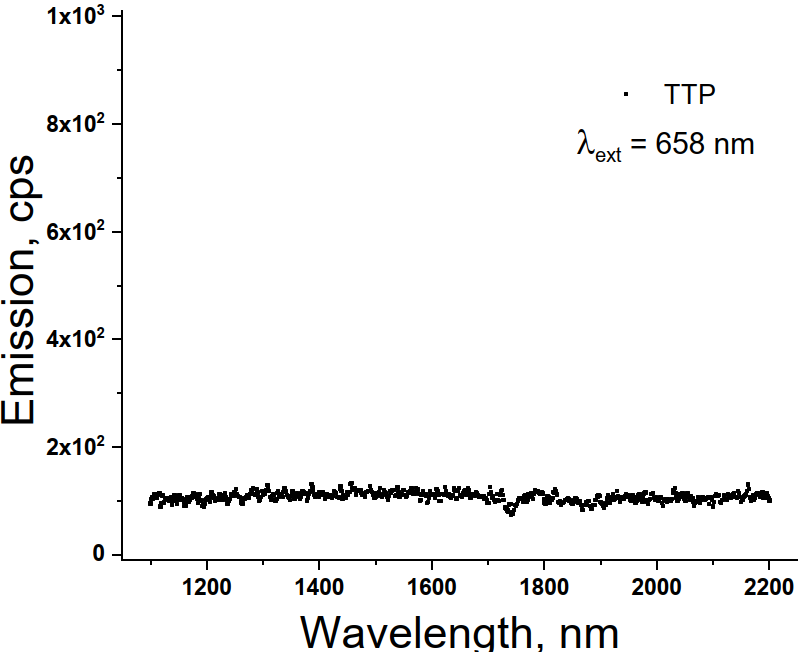


**Figure S9:** Emission spectrum of the metal-free TTP at 77K. The emission is registered by infrared fibre TE-cooled spectrometer. ***Experimental conditions***: sensitizer concentration TTP - 2×10^-5^M; nitrogen temperature, 77K; solvent: 2-Methyltetrahydrofuran; sample thickness – 1000 µm; single mode laser diode; λ_exc_ = 658 nm; optical registration – *via* fiber spectrometer, Hamamatsu, TG-cooled NIR II / C9914GB.
